# Reducing Blood Culture Contamination in Adult Patients with Cancer Presenting to the Emergency Department

**DOI:** 10.1017/ash.2024.302

**Published:** 2024-09-16

**Authors:** Kim Nguyen, Amy Spallone, Adriana Wechsler, Karim Samir Abdeldaem, Maryam Zaghian, Mymimilami Santos, Holly Ayers, Micah Bhatti, Goley Richardson, Brian Cameron

**Affiliations:** The University of Texas MD Anderson Cancer Center; University of Texas MD Anderson.org

## Abstract

**Background:** Infection is one of the most common complications of cancer and cancer treatment. Most patients admitted for fever or infection come through the Emergency Department (ED), which is a primary site for blood culture collection. Contamination of blood cultures complicates the diagnoses, compromises quality of care, leads to unnecessary antibiotic exposure and increases financial burdens. It may also lead to unnecessary removal of central venous access devices or delay of critical therapy or procedures. At our institution, the contamination rate of blood cultures drawn in the ED was over twice that of the remainder of the hospital (2.8 versus 0.8), prompting this quality improvement project. Unlike on hospital floors, nurses, instead of phlebotomists, draw most blood cultures due to the urgency of managing suspected sepsis. Our aim was to decrease the ED contamination rate by 20 percent after the first PDSA cycle, and ultimately bring it on par with the remainder of the hospital. **Methods:** First, we compared ED contamination rates versus other hospital inpatient floors and outpatient centers over a three-month period. We then evaluated the contamination rates of ED nurses versus ED phlebotomists and peripheral versus central line blood draws. Process mapping and fishbone analysis helped identify practices contributing to higher contamination rates. Key drivers of these practices were diagrammed, and potential interventions were ranked on a prioritization matrix. **Results:** We identified use of alcohol rather than chlorhexidine swabs for peripheral disinfection and inconsistent techniques of blood draw by nurses as critical contributors to increased contamination rates in the ED. Our intervention was creating premade blood culture kits promoting the use of chlorhexidine swabs through availability and easy access in the fast-paced ED environment. Ten cubic centimeter (cc) syringes in the kits encouraged withdrawal of adequate blood samples in compliance with the 7-10 cc guideline. Designated nursing team leaders checked off ED nurses at the bedside, implementing education and adherence in using the blood culture collection kits. The average number of blood cultures in the emergency department was 1,400. A reduction in blood culture contamination from 2.46 percent to 1.89 percent was seen after two months. **Conclusions:** A guideline-driven, standardized blood culture collection process followed by ED nurses is vital to reducing blood culture contamination. Chlorhexidine is necessary to maintain the lowest contamination rates. Readily available premade blood culture kits improve compliance with materials and techniques associated with best practices.

